# Evaluation of Clinical Outcomes of an Isofocal Optic-Design Intraocular Lens with Double C-Loop Haptics in Eyes with Glaucoma

**DOI:** 10.3390/jcm15145730

**Published:** 2026-07-22

**Authors:** Takayuki Akahoshi

**Affiliations:** Department of Cataract and Refractive Surgery, Nihonbashi Cataract-Clinic, Tokyo 103-0022, Japan; eye@phaco.expert

**Keywords:** intraocular lens, isofocal, double C-loop, glaucoma, cataract

## Abstract

**Background/Objectives:** To assess the refractive and visual outcomes after implantation of an isofocal optic-design intraocular lens (IOL) with double C-loop haptics following cataract surgery in eyes with primary open-angle glaucoma (POAG). **Methods:** This was a prospective, open-label, interventional exploratory study that considered POAG eyes undergoing cataract surgery with implantation of the Isopure Serenity (Beaver-Visitec International, Inc. [BVI], Waltham, MA, USA) IOL. Primary outcomes measured included manifest refraction, monocular uncorrected distance visual acuity (UDVA), corrected distance visual acuity (CDVA), uncorrected intermediate visual acuity (UIVA) and distance-corrected intermediate visual acuity (DCIVA) at 80 and 66 cm, uncorrected near visual acuity (UNVA), and distance-corrected near visual acuity (DCNVA) at 40 cm, and photopic binocular defocus curve were evaluated 3 months after surgery. **Results:** In total, 22 eyes of 11 patients (nine women, mean age of 68.3 ± 8.3 years) with POAG were enrolled and completed the clinical study. The study found that 100% of the eyes were within ±0.50 D and 86.36% of the eyes showed a postoperative refractive cylinder ≤ 0.25 D. The mean spherical equivalent and refractive cylinder were −0.06 ± 0.30 D and −0.13 ± 0.33 D, respectively. It was also shown that 86.36% and 100% of the eyes had 20/20 or better UDVA and CDVA, respectively. Furthermore, 95.45% and 77.27% of eyes presented a monocular DCIVA value of ≥20/32 at 80 and 66 cm, respectively, and 68.18% of eyes presented a monocular DCNVA value of ≥20/40. The mean monocular CDVA, DCIVA at 80 cm, DCIVA at 66 cm, and DCNVA were −0.09 ± 0.04, 0.12 ± 0.07, 0.21 ± 0.07 and 0.32 ± 0.07 logMAR, respectively. The binocular defocus curve showed good visual acuity at distance and intermediate vision. **Conclusions:** The isofocal Isopure Serenity IOL can be implanted in eyes with POAG after cataract surgery, achieving a satisfactory visual performance. Our results suggest that this IOL model is a viable option for selected patients with controlled early/moderate POAG and without central visual field involvement diagnosed with cataracts.

## 1. Introduction

The use of multifocal intraocular lenses (IOLs) in glaucoma and ocular hypertensive patients with no disk or visual field damage who have been stable may be candidates for multifocal IOLs, despite being a controversial topic due to the possible reduction in contrast sensitivity [1]. In addition, it should be taken into account that the choice of a specific IOL in glaucoma patients with cataracts should also consider the possible effect on patient’s visual acuity and visual field. Multifocal or enhanced depth of focus (EDOF) IOLs may be used in patients with milder forms of glaucoma [1]. A recent editorial about insights in glaucoma has pointed out that the evolution of IOL technology allows cataract surgeons to offer our patients solutions for spectacle independence after surgery. EDOF IOLs are best-suited for stable glaucoma patients with mild disease who have preserved central visual fields and contrast sensitivity [2]. In the recent guidelines from the French Glaucoma Society and the Society of the French Intraocular Lens and Refractive Surgery Association, it has been considered that monofocal plus IOLs and certain EDOF IOLs may be viable options, provided that specific precautions are taken [3]. These guidelines conclude that the indications for premium IOLs in glaucoma patients should include rigorous, individualized selection, along with comprehensive patient counseling. Enhanced IOLs aimed to provide improved intermediate vision while maintaining the benefits of a standard monofocal IOL. These lenses may be related to less photic phenomena and reduced contrast sensitivity and are therefore also a good option for glaucoma patients.

Previous retrospective, prospective and ambispective studies have been published on the use of EDOF and enhanced monofocal IOLs in patients diagnosed with glaucoma [4,5,6,7,8,9,10,11,12,13,14], concluding that these lenses may generally be recommended in these patients as an effective and safe solution in cataract surgery. The Isopure Serenity (Beaver-Visitec International, Inc. [BVI], Waltham, USA) IOL is an aspherical lens aiming to provide good visual acuity at distance with improved intermediate vision whilst inducing minimal photic phenomena. Recent clinical studies [15,16] have shown that patients implanted with this IOL showed good visual performance at distance with functional intermediate vision and accurate refractive outcomes. However, to the best of our knowledge, this study is the first in the literature to report the clinical results of this IOL in patients with glaucoma. Thus, the purpose of the current study is to evaluate the visual and refractive outcomes of the Isopure Serenity IOL in patients with primary open-angle glaucoma (POAG) undergoing cataract surgery.

## 2. Materials and Methods

We conducted a prospective open-label clinical exploratory study at our institution, the Nihonbashi Cataract Clinic located in Tokyo (Japan), between June 2024 and February 2026. This study adhered to the principles of the Declaration of Helsinki and received ethical approval from the Nihonbashi Cataract Clinic Review Board. Prior to the procedure, all participants provided written informed consent for both the surgery and the subsequent use of their de-identified data for research and statistical purposes. Inclusion criteria included patients with visually significant cataracts and well controlled early or moderate POAG, who had been using medical treatment for over 6 months and had been diagnosed with no central visual field defect. Patients’ optic nerve OCT images and Humphrey visual field test results were reviewed prior to surgery. Exclusion criteria included being age 18 and under, having uncontrolled, severe, or secondary open-angle glaucoma or angle-closure glaucoma. Glaucoma medication included Timoptol (Santen, Osaka, Japan), Xalatan (Pfizer, Tokyo, Japan), Tapcom (Santen, Osaka, Japan), Travatan (Alcon, Fort Worth, TX, USA), Rescula (Novartis, Basel, Switzerland), Tafluprost (Santen, Osaka, Japan), Azopt (Novartis, Basel, Switzerland), Latacom (Duopharma, Kuala Lumpur, Malaysia), or Azarga (Novartis, Basel, Switzerland) (only one type of medication per eye).

All eyes received either a toric or non-toric hydrophobic isofocal Isopure intraocular lens (IOL). This lens is made of GFY (a hydrophobic acrylic material with a refractive index of 1.53 and an Abbe number of 42) and features both blue light and UV filters. It has a 6.00 mm optical zone and an overall diameter of 11.4 mm. The haptic design incorporates a posterior angulated POD double C-loop with Ridge-Tech technology. Spherical IOL powers were available in +0.50 D increments from +10 to +30 D and in +1 D increments from +31 to +35 D for spherical lenses, and the cylindrical power was 1.00, 1.50, 2.25, 3.00, 3.75, 4.50, 5.25, and 6.00 D (at the IOL plane). The same experienced surgeon (TA) performed all the surgeries using the Centurion Phacoemulsificator machine (Alcon Labs, Fort Worth, TX, USA) by means of the Phaco Prechop technique [16,17,18,19,20]. The Akahoshi Intra-operative Axis Marker (ASICO AE-2933, ASICO, Nagoya, Japan) was used for marking the toric axis in toric IOLs. In eyes with corneal astigmatism, a toric IOL model was used. The Barrett Universal II formula was used for IOL power calculation considering that the target refraction was emmetropia in all eyes. Optical biometry was used to measure the main ocular parameters (axial length, anterior chamber depth, lens thickness, K1, K2, axis K1, axis K2, and white-to-white distance) by means of the IOLMaster 700 instrument (Carl Zeiss Meditec AG, Jena, Germany).

Preoperatively, patient demographics, previous ocular history and medications were collected. In addition, ocular biometry, visual acuity, intraocular pressure, specular microscopy and refraction were also obtained in all cases. Following surgery, visual outcomes were evaluated at the 3-month postoperative visit. The recorded metrics included monocular uncorrected distance visual acuity (UDVA), corrected distance visual acuity (CDVA), uncorrected intermediate visual acuity (UIVA), and distance-corrected intermediate visual acuity (DCIVA) measured at both 80 and 66 cm. Additionally, uncorrected near visual acuity (UNVA) and distance-corrected near visual acuity (DCNVA) were assessed at 40 cm. All visual acuity testing was performed using Sloan ETDRS charts (Precision Vision, Woodstock, IL, USA) on a logMAR scale. Taking into account that this lens offers intermediate vision, a binocular defocus curve was also performed to determine the visual acuity obtained at different vergences (from −4 D to +1 D, in 0.50 D steps). Manifest refraction at the last visit and a vector analysis (double-angle plot tool [21] based on the preoperative corneal astigmatism and the refraction at the last visit) were also obtained to assess the accuracy of the procedure. Adverse events, if any, were also recorded. For all parameters recorded, the average, standard deviation and ranges were obtained using Excel software (2019, version 16.43, Microsoft Corporation, Redmond, WA, USA), and standard graphs for reporting IOL surgery outcomes were created [22].

## 3. Results

A total of 22 eyes of 11 patients implanted with the Isopure Serenity IOL participated in our clinical study. A total of 9 patients were female (81.8%). Preoperative characteristics and demographics of the patients are shown in Table 1. The mean patient age was 68.3 ± 8.3 years (ranging from 56 to 84 years). No surgical complications or adverse events related to the lens were reported in our cohort. A total of 21 IOLs were non-toric with a mean of 18.68 ± 3.19 D (ranging from 12.00 to 24.00 D) and one IOL toric with toric IOL power of 1.50 D.

**Figure 1:**
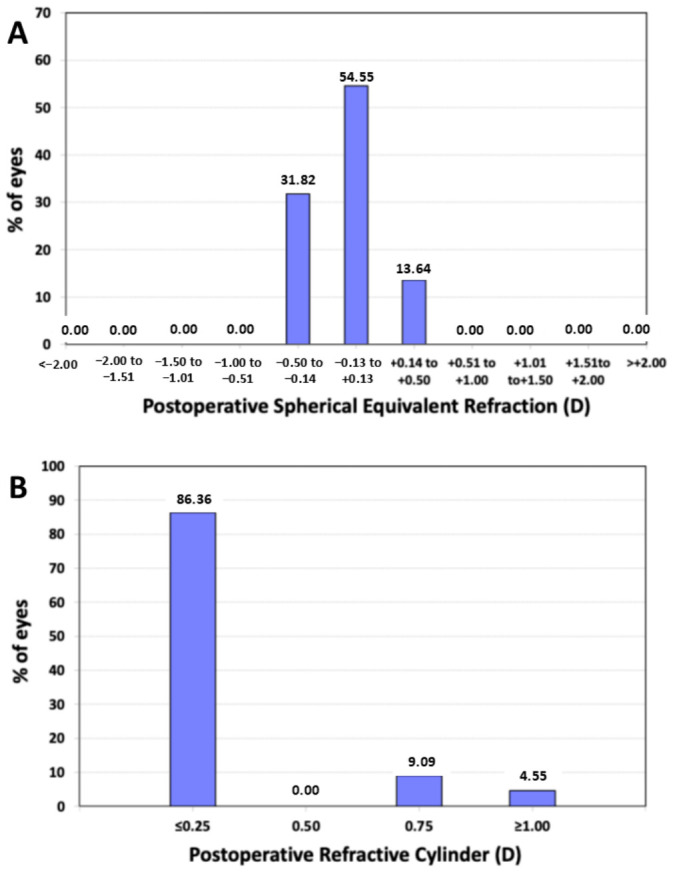
Accuracy in terms of spherical equivalent refraction (**A**) and refractive cylinder (**B**) post-Isopure Serenity intraocular lens implantation.

**Figure 2:**
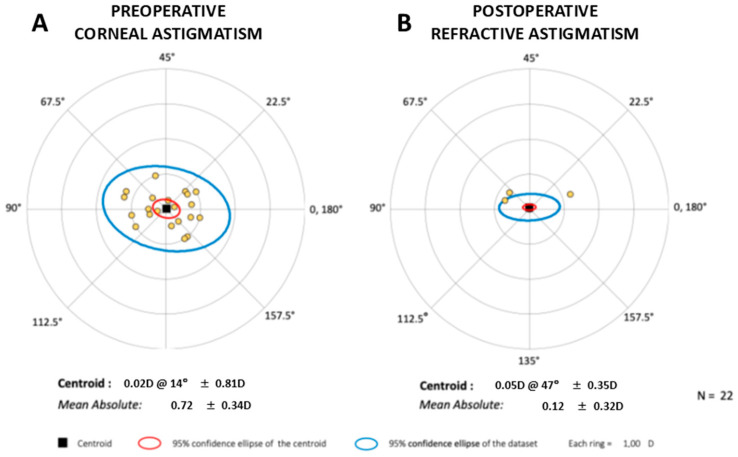
Double-angle plots for corneal astigmatism before the surgery (**A**) and refractive astigmatism (**B**) at 3 months post-Isopure Serenity intraocular lens implantation.

Standard graphs for IOL-based surgery were created. Figure 1 shows the accuracy of the intervention, showing the distribution of the spherical equivalent (Figure 1A) and the refractive cylinder (Figure 1B) after the surgery (3 months’ follow-up). Note that the highest percentage of eyes, 54.55% (n = 12 eyes), was for the range between ±0.13 D, followed by 31.32% (n = 7 eyes) for the −0.14 to −0.50 D range. It was also shown that 100% of the eyes were within ±0.50 D. At 3 months, the mean spherical equivalent and refractive cylinder were −0.06 ± 0.30 D (ranging from −0.50 to 0.50 D) and −0.13 ± 0.33 D (ranging from −0.50 to 0.50 D), respectively. A total of 86.36% of the eyes (n = 19 eyes) showed a postoperative refractive cylinder ≤ 0.25 D. In relation to the astigmatic vector analysis, we have created Figure 2, which shows the preoperative corneal astigmatism before the surgery, obtained from the optical biometer (Figure 2A) and the subjective refractive astigmatism postoperatively at 3 months (Figure 2B). The mean absolute of the corneal astigmatism before surgery was 0.72 ± 0.34 D and that of the subjective refractive astigmatism was 0.12 ± 0.32 D after the IOL implantation at 3 months. Note that there is a reduction in the spread of the dots, indicating its reduction post-surgery.

**Figure 3:**
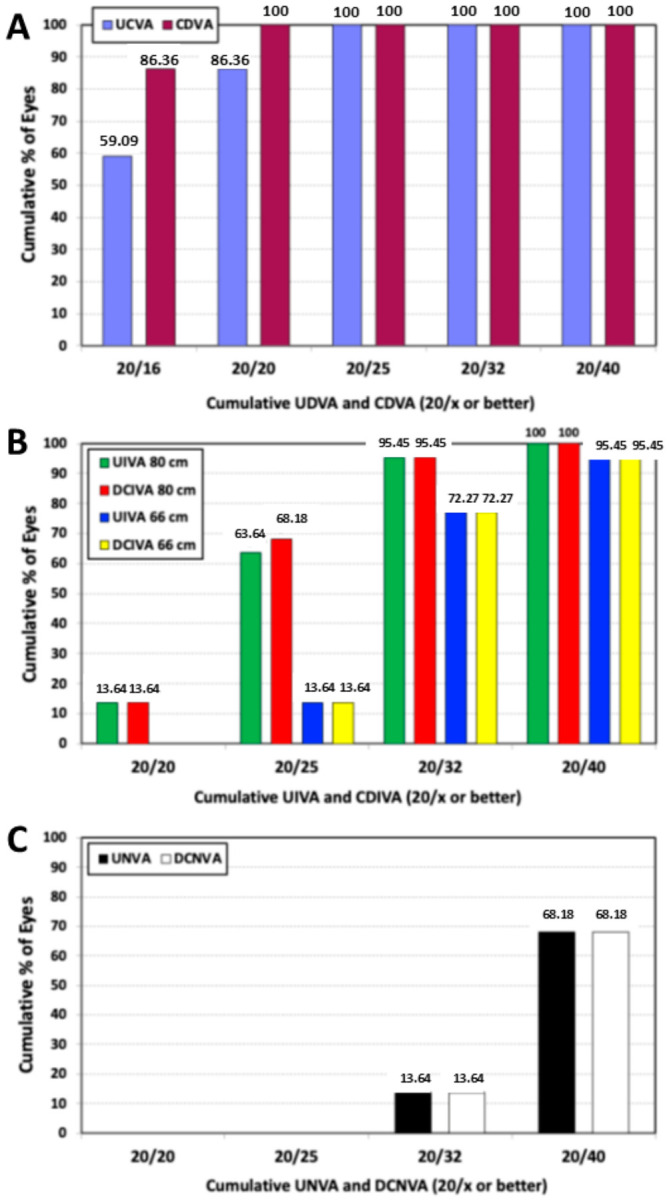
Cumulative percentage of eyes at 3 months post-surgery, with a given postoperative uncorrected distance visual acuity (UDVA) and corrected distance visual acuity (CDVA) (**A**), uncorrected intermediate visual acuity (UIVA) and distance-corrected intermediate visual acuity (DCIVA) at 80 and 66 cm (**B**), and uncorrected near visual acuity (UNVA) and distance-corrected visual acuity (DCNVA) at 40 cm (**C**).

In relation to visual acuity outcomes at different distances, Figure 3 was created. It shows the cumulative proportion of eyes at 3 months with a given UDVA and CDVA (Figure 3A), UIVA and DCIVA (Figure 3B) and UNVA and DCNVA (Figure 3C) values. At 3 months, 19 (86.36%) and 22 eyes (100%) had ≥20/20 UDVA and CDVA, respectively, with 22 eyes (100%) achieving ≥20/25 for both UDVA and CDVA. The mean values for UDVA and CDVA were −0.05 ± 0.06 and −0.09 ± 0.04 logMAR, respectively. Table 2 shows the mean values for both uncorrected and distance-corrected visual acuity measured at different distances. For intermediate vision (see Figure 3B), 21 eyes (95.45%) achieved ≥20/32 DCIVA at both 80 and 66 cm, with 22 eyes (100%) and 21 eyes (95.45%) achieving ≥20/40 DCIVA at 80 and 66 cm, respectively. The mean values for these two distances of DCIVA were 0.12 ± 0.07 and 0.21 ± 0.07 logMAR at 80 cm and at 66 cm, respectively, as detailed in Table 2. At near vision (see Figure 3C), three eyes (13.64%) had ≥20/32 UNVA and DCNVA, with 15 eyes (68.18%) achieving ≥20/40 UNVA and DCNVA. The mean DCNVA at 40 cm was 0.32 ± 0.07 logMAR as indicated in Table 2.

**Figure 4:**
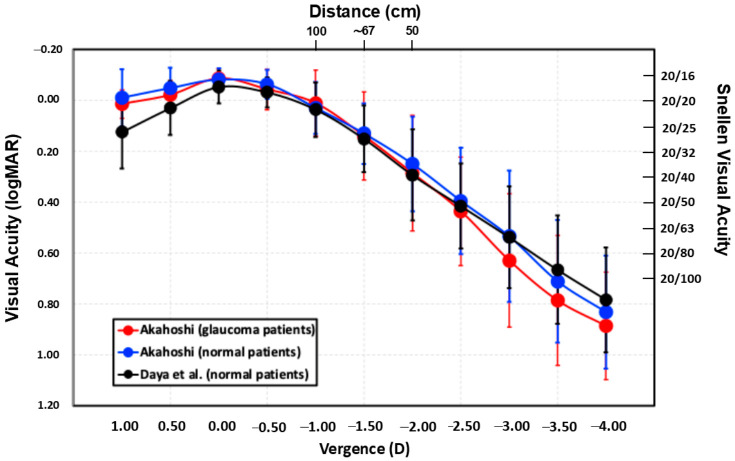
Mean binocular best distance-corrected visual acuity (logMAR) as a function of chart vergence from +1 D to −4 D at 3 months post-Isopure Serenity intraocular lens (IOL) implantation. Error bars: standard deviation. The right *Y*-axis shows Snellen feet acuity. Data for normal patients implanted with the same lens at 3 months post-surgery were included from the Akahoshi [15] (19 patients) and Daya et al. [16] (54 patients) studies.

To assess the outcomes of visual acuity at different defocus values, Figure 4 was plotted. It depicts the postoperative binocular through-focus, visual acuity (best-corrected) from +1 D to −4 D at 3 months (n = 8 patients). This graph also includes data of normal patients implanted with the same IOL at 3 months post-surgery from the Akahoshi [15] (n = 19 patients) and Daya et al. [16] (n = 54 patients) studies for comparative purposes (indirect descriptive comparison). As expected, there is a maximum value of visual acuity located at 0 D (distance vision) with a smooth reduction in acuity with negative values of vergence (closer distances).

## 4. Discussion

The current clinical study has assessed the refractive and visual outcomes of patients diagnosed with POAG implanted with the Isopure Serenity IOL after cataract surgery. In this type of eye, our results reveal that this IOL provided an extended range of vision with accurate refractive outcomes. Specifically, our results reveal that 100% of the eyes were within ±0.50 D (Figure 1) with a mean spherical equivalent close to emmetropia and a mean refractive cylinder less than a quarter of diopter showing the refractive astigmatism correction (see Figure 2 for the concentration of the spots). The accuracy of the procedure agrees with that reported by the same IOL in normal patients as found in two previous studies [15,16]. Akahoshi [15] assessed 38 normal eyes with this IOL model at 3 months, obtaining 100% of eyes within ±1.00 D and 89.47% of eyes within ±0.50 D, with a mean postoperative spherical equivalent of 0.12 ± 0.35 D and a mean refractive cylinder of −0.11 ± 0.33 D. Daya et al. [16], with a large sample of 108 eyes in a multicenter study at 3 months, reported 79.63% of eyes within ±0.50 D and 98.15% of eyes within ±1.00 D, with an average refractive spherical equivalent and cylinder values of −0.06 ± 0.44 D and −0.37 ± 0.44 D, respectively. The design of this IOL aimed to provide a continuous range of vision offering good visual acuity at distance and intermediate vision. This is obtained in our visual acuity outcomes (see Table 2). The mean values for CDVA, DCIVA and DCNVA were −0.09 ± 0.04, 0.12 ± 0.07 and 0.32 ± 0.08 logMAR, respectively. The visual outcomes at different distances were consistent with those reported by the same IOL in normal patients as reported in the previous studies [15,16]. This is achieved by the design of the Isopure Serenity IOL based on an isofocal concept. Akahoshi [15] found mean values of −0.08 ± 0.05, 0.23 ± 0.13 (80 cm), 0.28 ± 0.13 (66 cm) and 0.35 ± 0.14 logMAR, respectively; and Daya et al. [16] −0.04 ± 0.05, 0.19 ± 0.10 (80 cm), 0.28 ± 0.11 (66 cm), and 0.40 ± 0.13 logMAR, respectively. The defocus curve, which also shows the change in visual acuity at different vergences, supports good vision at different distances (see Figure 4 for detailed values). This figure also depicts the values obtained in the previous two studies in normal eyes for comparative purposes, and it may be observed that the values were directly comparable among the three groups of patients (two groups of normal eyes [blue and black dots] and one group with POAG [red dots]). Thus, our results (based on eight patients) confirm that the refractive and visual acuity outcomes were good and comparable to those found in normal eyes implanted with this IOL model.

As introduced, EDOF IOLs are best-suited for stable glaucoma patients with mild disease who have preserved central visual fields and contrast sensitivity [2] and that monofocal plus IOLs and certain EDOF IOLs may be viable options, provided that specific precautions are taken [3]. It is worth discussing the outcomes of studies that analyze the use of EDOF or enhanced monofocal IOLs in patients diagnosed with glaucoma. For this purpose, Table 3 was created. This table shows a summary of peer-reviewed clinical studies analyzing the use of EDOF IOLs, specifically Tecnis Symfony (Johnson & Johnson, Santa Ana, CA, USA), AcrySof IQ Vivity (Alcon Labs, Fort Wort, TX, USA) and Lucidis (Swiss Advanced Vision, SAV-IOL SA, Neuchâtel, Switzerland), and the enhanced monofocal IOL Tecnis Eyhance (Johnson & Johnson, Santa Ana, CA, USA), in patients diagnosed with glaucoma. This table shows the authors, IOL model used, number of eyes and patients of the sample, type of study, type of glaucoma and conclusions obtained by the different authors. As indicated in the introduction, no previous studies with the Isopure Serenity IOL have been carried out in glaucoma patients, making our study the first, and therefore direct comparison with previous literature on the same lens is not possible. However, we can discuss with reference to the outcomes obtained using other IOL models.

### 4.1. EDOF Intraocular Lenses

The AcrySof IQ Vivity IOL was assessed in four studies [4,6,13,14]. Kerr et al. [4] carried out an observational retrospective comparative study with this IOL and the Clareon/SN6ATx/SN60WF (Alcon Labs, Fort Wort, TX, USA) in 29 patients with early glaucoma, 32 eyes for the Vivity group and 26 eyes for the monofocal group. LogMAR UDVA, CDVA, UIVA and UNVA were 0.12 ± 0.15, 0.01 ± 0.09, 0.06 ± 0.16, and 0.29 ± 0.10 for the Vivity group, respectively, and 0.10 ± 0.13, 0.00 ± 0.10, 0.39 ± 0.10 and 0.55 ± 0.18, for the monofocal group, respectively. The differences between both groups were statistically significant only for intermediate and near visual acuity (*p* < 0.001). The accuracy (spherical equivalent) was similar between both groups: −0.35 ± 0.34 D and −0.26 ± 0.42 D (*p* = 0.887). These authors also analyzed photic phenomena, reporting no significant difference in the incidence between both groups (*p* > 0.2), and patient satisfaction with spectacle-free vision, being higher in the EDOF group for distance, intermediate and near vision (*p* < 0.05). They concluded that the bilateral use of the AcrySof IQ Vivity lens provided excellent distance vision and better intermediate and near vision than monofocal IOLs in patients with early glaucoma and that this lens can be considered to correct presbyopia and reduce the dependence on glasses of patients with ocular hypertension and stable early glaucoma undergoing cataract surgery. Ferguson et al. [6] enrolled 26 patients diagnosed with mild stage (pre-perimetric) open-angle glaucoma, who were implanted with the Vivity lens and analyzed at 4 months post-surgery. They obtained a mean binocular UDVA of 0.03 ± 0.12 logMAR with 85% of the patients achieving UDVA ≥ 20/25 and 65% achieving UDVA ≥ 20/20. The mean CDVA was −0.06 ± 0.07 logMAR and 96% were ≥20/20. The mean binocular UIVA was 0.17 ± 0.12 logMAR with 77% of patients achieving UIVA ≥ 20/32 and 46% were ≥20/25, and the mean binocular UNVA was 0.31 ± 0.17 logMAR with 65% achieving UNVA ≥ J3 and 46% achieving ≥J2. 83% and 96% had a mean spherical equivalent within ±0.50 D and ±1.00 D, respectively (mean −0.27 ± 0.35 D). The authors concluded that this lens can be safely implanted in eyes with mild, pre-perimetric open-angle glaucoma with favorable UDVA and UIVA. Liu et al. [13] analyzed 31 patients with ocular hypertension and well controlled mild glaucoma undergoing cataract surgery with Vivity IOL implantation. At 3 months post-surgery, mean UDVA, UIVA and UNVA were 0.02, 0.07 and 0.267 logMAR, respectively. A total of 96% of eyes had UDVA ≥ 20/25, 84% of eyes had UIVA ≥ 20/30 and 63% of eyes had UNVA ≥ 20/40. These authors concluded that this lens is an option for patients with ocular hypertension or early glaucoma in the short term. These patients experienced significant improvement in spectacle-free distance and intermediate vision, and greater spectacle independence at near. They also indicated an acceptable dysphotopsia profile and reduced glare, emphasizing the potential of non-diffractive EDOF IOLs in enhancing the overall visual experience for patients with mild, well controlled glaucoma. And, recently, Urcola et al. [14], in an ambispective study with 36 patients with POAG, found binocular CDVA, DCIVA, CDNVA of 0.00 ± 0.12, 0.16 ± 0.14, and 0.24 ± 0.11 logMAR, respectively, with 86.11% of eyes within ±0.50 D and 95.83% within ±1.00 D (mean spherical equivalent of −0.27 ± 0.33 D). They also reported that 97.06% of patients never reported shadow areas. They concluded that the Vivity IOL seems to provide good visual acuity at far, intermediate and near vision, with an adequate contrast sensitivity, defocus curve, and low rate of visual disturbances with high visual satisfaction in patients with mild and stable POAG.

The Tecnis Symfony IOL was analyzed in three studies [7,8,10]. Bissen-Miyajima et al. [7] prospectively compared the Symfony lens with a monofocal IOL in 22 and 24 eyes with mild-to-moderate POAG with cataracts, respectively. They analyzed the mean deviation in the visual field and obtained no significant differences between both groups (mean −2.76 dB and −4.21 dB, respectively). They found that CDVA and contrast sensitivities in the EDOF group were not inferior to those of eyes with monofocal IOLs (mean logMAR CDVA of −0.17 and −0.08, respectively) with 100% of eyes showing a cumulative CDVA ≥ 20/20 in the EDOF group. They concluded that the visual function of eyes with mild-to-moderate primary open-angle glaucoma implanted with the EDOF lens was not inferior to that found in eyes implanted with the monofocal lens. The same year, these authors published a case series of 10 patients (16 eyes) with normal tension glaucoma with no central visual field defects implanted with this IOL model and further concluded, at 3 months’ follow-up, that the visual function of these patients was almost comparable to those of normal eyes with the same IOL model, permitting the use of this IOL in controlled normal tension glaucoma eyes [8]. They also aimed to investigate the association of perimetry parameters with visual acuity and contrast sensitivity and concluded that the visual function of this type of eye implanted with the EDOF lens was associated with perimetry parameters in high spatial frequency contrast sensitivity, which was different from that of POAG eyes implanted with monofocal lenses [10].

The Lucidis IOL (hybrid refractive EDOF IOL) was analyzed by Dessouky et al. [12] in early-to-moderate POAG eyes (n = 42). They reported mean UDVA, UIVA, UNVA, CDVA and CDNVA of 0.05 ± 0.09, 0.07 ± 0.08, 0.08 ± 0.10, 0.01 ± 0.06 and 0.06 ± 0.08 logMAR, respectively. They found 74% and 90% of eyes within ±0.50 D and within ±1.00 D, respectively, with a mean spherical equivalent of −0.19 ± 0.46 D. They also measured contrast sensitivity, obtaining a mean value of 1.56 ± 0.11 logMAR. The authors indicated that the refractive hybrid design of this lens could be of added benefit in patients with glaucoma based on the adequate range of visual acuity obtained, without compromising contrast sensitivity or causing photic phenomena in patients with early-to-moderate POAG.

### 4.2. Enhanced Monofocal Intraocular Lens

The IOL Tecnis Eyhance was used in three studies [5,9,11]. Nam et al. [5] prospectively compared this lens with a standard monofocal (n = 38 eyes) one in eyes with medically controlled early open-angle glaucoma without central visual field defects (n = 34 eyes) at 3 months post-surgery. They found that the Tecnis Eyhance IOL provided better UIVA than the standard monofocal IOL (*p* = 0.003) but similar UDVA, CDVA, and UNVA. They also found that the enhanced monofocal IOL provided better satisfaction (*p* = 0.019) and lower spectacle dependence (*p* = 0.004) than the standard monofocal IOL for intermediate vision, with similar visual field and contrast sensitivity outcomes. They concluded that enhanced monofocal IOLs are recommended for patients with open-angle glaucoma, as they provide better intermediate vision, higher satisfaction, and lower dependence on spectacles than standard monofocal IOLs, without worsening other visual outcomes. Tojo et al. [9] retrospectively analyzed this model in all types of glaucoma eyes (n = 58) comparing with eyes implanted with a non-enhanced IOL (n = 46). They measured the value of the foveal threshold using a Humprey visual field analyzer before and after cataract surgery and found that they did not significantly differ as a function of the IOL type implanted (*p* = 0.673). In this sense, they agreed with Nam et al. [5] and concluded that the use of enhanced monofocal IOLs in glaucoma patients did not result in any apparent loss of sensitivity compared with non-enhanced monofocal IOL implantation. Chung et al. [11] also reported similar conclusions. They prospectively analyzed 60 eyes of 30 patients with pre-perimetric glaucoma and 60 eyes of 30 patients without retinal nerve fiber layer defects, being compared at 3 months post-surgery. They found no difference in binocular UDVA, UIVA and UNVA, proportion of severe or very severe photic phenomena (glare and halos) and overall satisfaction. Thus, they concluded that the bilateral use of this IOL model in patients with pre-perimetric glaucoma showed commensurate clinical outcomes and could be considered a feasible alternative.

In general, our results agree with these previous studies using different IOL models in glaucoma patients, with some studies showing comparable mean visual acuities at different distances and that these lenses can be used in this type of patient to provide an extended range of vision without reducing contrast sensitivity. Thus, the Isopure Serenity IOL model can be added to this armamentarium for cataract surgery in order to provide high patient satisfaction and reduce spectacle dependence.

Our preliminary study has several limitations that should be pointed out. First, we had neither a control group of POAG patients implanted with monofocal IOL, nor a control group of normal eyes implanted with the Isopure Serenity IOL. However, we included the outcomes obtained in previous studies, including ours, in order to properly compare the outcomes of normal patients with this new IOL. Second, our analysis was done at 3 months with a limited sample and considering both eyes of the same patient, meaning long-term assessment with a large sample would be required to confirm these preliminary outcomes. Other metrics related to visual performance such as contrast sensitivity or perimetry data would also be worth including in future studies in order to control possible degradation of contrast sensitivity. Also, patient-reported outcomes questionnaires would add clinical value about the performance of the lens in these patients.

## 5. Conclusions

In conclusion, our exploratory study shows that the new isofocal Isopure Serenity IOL with double C-loop haptics results in accurate refractive outcomes with excellent visual performance for distance vision and functional intermediate vision in patients with POAG. Note that the findings reported apply to carefully selected patients with controlled early/moderate glaucoma and without central visual field involvement. This procedure is a useful and viable option for this type of patient and may be considered by cataract surgeons as an alternative to monofocal or multifocal IOLs.

## Figures and Tables

**Table 1:** Preoperative and demographic characteristics of participants shown as means, standard deviations (SD) and ranges.

	Isopure Serenity IOL
Patients)	11
Eyes (n)	22
Sex (m/f)	2/9
Age (y)	68.3 ± 8.3 (56 to 84)
Sphere (D)	−1.35 ± 3.28 (−8.50 to 3.50)
Refractive cylinder (D)	−1.03 ± 0.92 (−3.25 to 0)
Spherical equivalent (D)	−1.87 ± 3.23 (−9.25 to 2.38)
Intraocular pressure (mmHg)	13.65 ± 2.44 (10 to 18)
CDVA (logMAR)	0.11 ± 0.15 (−0.08 to 0.40)
K1 (D)	42.64 ± 3.98 (34.50 to 46.50)
K2 (D)	43.35 ± 3.94 (35.25 to 47.25)
Axial length (mm)	24.94 ± 2.27 (22.76 to 29.15)
Anterior chamber depth (mm)	3.11 ± 0.11 (2.42 to 3.64)
Lens thickness (mm)	4.50 ± 0.49 (3.66 to 5.18)
White-to-white (mm)	11.77 ± 0.37 (11.20 to 12.50)
Spherical IOL power (D)	18.68 ± 3.19 (12.00 to 24.00)

CDVA: corrected distance visual acuity; K: keratometry; IOL: intraocular lens power.

**Table 2:** Postoperative logMAR monocular visual acuity values for eyes implanted with the Isopure Serenity lens shown as means, standard deviations (SD).

	Isopure Serenity IOL
UDVA	−0.05 ± 0.06 (−0.10 to 0.05)
CDVA	−0.09 ± 0.04 (−0.10 to 0.00)
UIVA (80 cm)	0.12 ± 0.07 (0.00 to 0.30)
DCIVA (80 cm)	0.12 ± 0.07 (0.00 to 0.30)
UIVA (66 cm)	0.21 ± 0.07 (0.10 to 0.40)
DCIVA (66 cm)	0.21 ± 0.07 (0.10 to 0.40)
UNVA (40 cm)	0.32 ± 0.07 (0.20 to 0.40)
DCNVA (40 cm)	0.32 ± 0.07 (0.20 to 0.40)

UDVA: uncorrected distance visual acuity; CDVA: corrected distance visual acuity; UIVA: uncorrected distance intermediate visual acuity; DCIVA: distance-corrected intermediate visual acuity; UNVA: uncorrected distance near visual acuity; DCNVA: distance-corrected near visual acuity.

**Table 3:** Peer-reviewed case reports and clinical studies analyzing the use of extended depth of focus (EDOF) or enhanced monofocal (EM) intraocular lenses (IOLs) in patients with glaucoma.

Authors (Year)	IOL Model	Eyes (Patients)	Study Type	Patients	Follow-Up (Months)	Conclusions
Kerr et al. [4] (2023)	AcrySof IQ Vivity	32 (16)	Retrospective	Early glaucoma	3 weeks to 3 months	Bilaterally implanted EDOF IOLs provided excellent distance vision and better intermediate and near vision than monofocal IOLs in patients with early glaucoma.
Nam et al. [5] (2023)	Tecnis Eyhance (ICB00)	34 (34)	Prospective	Medically controlled early OAG without central VF defects	3	EM IOLs are recommended for patients with OAG because they provide better intermediate vision, higher satisfaction, and lower dependence on spectacles than standard monofocal IOLs, without worsening other visual outcomes.
Ferguson et al. [6] (2023)	AcrySof IQ Vivity AcrySof IQ Vivity Toric *	52 (26)	Prospective	Mild OAG based on the AAO Preferred Practice Pattern guidelines	4	EDOF IOLs can be safely implanted in eyes with mild, pre-perimetric OAG with favorable uncorrected distance and intermediate visual acuity. The contrast sensitivity measurements were favorable and the subjective questionnaire revealed satisfactory spectacle independence and patient satisfaction.
Bissen-Miyajima et al. [7] (2023)	Tecnis Symfony (ZXR00V and ZXV150-375)	22 (22)	Prospective	Mild-to-moderate POAG	3	The visual function of EDOF IOLs in eyes with mild-to-moderate POAG was not inferior to that of monofocal IOLs.
Bissen-Miyajima et al. [8] (2023)	Tecnis Symfony (ZXR00V and ZXV150-375)	16 (10)	Retrospective	NTG	3	Postoperative visual functions of NTG patients with EDOF IOLs were almost comparable to those of normal eyes with the same IOLs, which demonstrated that the use of EDOF IOLs for controlled NTG eyes would be permissible.
Tojo et al. [9] (2024)	Tecnis Eyhance (DIB00V)	58 (32)	Retrospective	All types of glaucoma	1–3	Implantation of EM IOLs in glaucoma patients did not result in any apparent loss of sensitivity compared with non-EM lens implantation.
Bissen-Miyajima et al. [10] (2024)	Tecnis Symfony (ZXR00V, ZXV150-375 and ZXW150-375)	22 (22)	Prospective	POAG	3	The visual function of POAG eyes with EDOF IOLs was associated with perimetry parameters in high spatial frequency contrast sensitivity, which was different from that of POAG eyes with monofocal IOL.
Chung et al. [11] (2024)	Tecnis Eyhance (ICB00)	60 (30)	Prospective	Patients with bilateral RNFL defects and no glaucomatous VF defect in both eyes	3	Bilateral implantation of an EM IOL with enhanced intermediate function in patients with pre-perimetric glaucoma demonstrated commensurate clinical outcomes and could be considered a feasible alternative.
Dessouky et al. [12] (2024)	Lucidis	42 (28)	Retrospective	Early-to-moderate POAG	3	EDOF IOL is effective and safe in patients with cataracts and early-to-moderate POAG requesting spectacle independence.
Liu et al. [13] (2025)	AcrySof IQ Vivity AcrySof IQ Vivity Toric	55 (31)	Prospective	OHT and well controlled mild glaucoma	3	EDOF lens improves distance and intermediate spectacle-free visual function in patients with OHT and well controlled glaucoma. The findings highlight significant improvements in visual acuity, reduced glare, enhanced spectacle independence, and improved visual performance in different lighting conditions.
Urcola et al. [14] (2025)	AcrySof IQ Vivity	72 (36)	Ambispective	POAG	1.26–20.13	EDOF IOL seems to provide good visual outcomes at distance, intermediate, and near vision, with an adequate contrast sensitivity, defocus curve, a low rate of visual disturbances and high visual satisfaction in patients with mild and stable POAG.

Hodapp–Parish–Anderson classification of visual field loss included; OAG: open-angle glaucoma; VF: visual field; * with trabecular micro-bypass stent device (iStent inject) concomitant implanted at the time of cataract surgery; POAG: primary open-angle glaucoma; NTG: normal tension glaucoma; RNFL: retinal nerve fiber layer; OHT: ocular hypertension.

## Data Availability

This paper presents all the resultant statistical data from the study. Datasets produced and analyzed by the study are available upon request.

## Abbreviations

The following abbreviations are used in this manuscript:

IOL	Intraocular lens
EDOF	Enhanced depth of focus
POAG	Primary open-angle glaucoma
OCT	Optical coherence tomography
UDVA	Uncorrected distance visual acuity
CDVA	Corrected distance visual acuity
UIVA	Uncorrected intermediate visual acuity
DCIVA	Distance-corrected intermediate visual acuity
UNVA	Uncorrected near visual acuity
DCNVA	Distance-corrected near visual acuity
